# The Role of Classroom Contexts on Learners’ Grit and Foreign Language Anxiety: Online vs. Traditional Learning Environment

**DOI:** 10.3389/fpsyg.2022.869186

**Published:** 2022-03-07

**Authors:** Beibei Zhao

**Affiliations:** School of Jiayang/Foundation Studies/International Education, Zhejiang Shuren University, Hangzhou, China

**Keywords:** foreign language anxiety, grit, online learning, traditional learning, learning environment

## Abstract

This review aimed at exploring the related investigations on the effects of online and traditional learning contexts on English as a foreign language (EFL) learners’ grit and foreign language anxiety (FLA). Studies have verified the relationship between learners’ grit and academic performance in online learning contexts. However, there is a need for studying the effect of face-to-face learning and face-to-screen learning on learners’ grit. On the other hand, studies have shown that classroom context is a mediating variable in the relationship between grit and FLA. Furthermore, few studies have been done on the effect of traditional classroom contexts and online learning contexts on learners’ FLA. Most studies showed that online learning contexts create more FLA. There are some reasons such as ambiguity of contexts, lack of feedback, lack of opportunities for communication, type of employed applications, cognitive load, technophobia, and reduction in enthusiasm which arouse learners’ anxiety in an online learning environment. In the end, the pedagogical implications are expounded to promote the learners’ grit and diminish anxiety for better performance. This review also provides some suggestions for further research to clarify our perspective on positive and negative emotional variables.

## Introduction

Foreign language learners’ attempts have been affected by these variables, which make language learners suffer from communication apprehension when communicating in a foreign language for different purposes. Learners’ negative emotional constructs such as anxiety, boredom, burnout, and apprehension have been studied in more detail (MacIntyre, 2017; Fathi and Derakhshan, 2019; Li et al., 2020; Derakhshan et al., 2021; Li and Dewaele, 2021). After the emergence of positive psychology, many positive psychologists have considered learners’ strengths to improve their learning outcomes (MacIntyre et al., 2019; Wang and Derakhshan, 2021; Zheng et al., 2021a,b).

However, this review tends to reexamine the studies about the positive and negative constructs like grit and anxiety in traditional and online contexts, respectively. This review hopes to assist language learners to process language better in their minds in both online and traditional contexts. However, the effect of online and conventional environments on learners’ grit and anxiety can shed light on positive psychology and negative emotional constructs. Exploration of this study will be significant in the language learning education era.

## Literature Review

### The Concept of Grit

Grit, as a critical notion, has drawn the attention of educational investigators (Gray and Mannahan, 2017). According to Duckworth (2016), grit, as a non-cognitive construct, is more important than talent in academic achievement. Cross (2014) described grit as tolerating difficulties though preserving the wish for long-term purposes. Duckworth and Quinn (2009) also pointed out that grit is “a passion and perseverance to accomplish long-term goals whatever the obstacles and no matter how long it may take” (p. 541). Duckworth et al. (2007) classified this higher-order concept into two components: steadiness in interests and persistence in effort despite difficulties. Von Culin et al. (2014) also argued that gritty learners have willpower, perseverance, capability to determine well-defined objectives, endurance, and flexibility in facing difficulties in their academic life. Karlen et al. (2019) also highlighted these components in Grit Scale as a measurement. Recently, some studies have been done on grit as a relevant factor for predicting learners’ language success (Robins, 2019). Lee and Drajati (2019) also mentioned that learners require more grit to initiate foreign language communication. Regarding the relationship between grit and emotions, the investigations have approved a positive correlation between grit and passion (Sigmundsson, 2021), life satisfaction (Li et al., 2018), motivation (Muenks et al., 2018), and wellbeing (Lan and Moscardino, 2019). Changlek and Palanukulwong’s (2015) study revealed that learners’ grit is positively associated with motivations and negatively correlated with foreign language anxiety (FLA). Liu and Wang (2021) found out that grit, foreign language achievement, and enjoyment are significantly associated, and they have a significant negative correlation with FLA. Moreover, their study revealed mediating roles of anxiety and enjoyment in the correlation between grit and language learning achievement.

### The Concept of Foreign Language Anxiety

Gardner and MacIntyre (1993) pointed out that “Anxiety is fear or apprehension occurring when a learner is expected to perform in the second or foreign language” (p. 59). They linked it to the stimulation of the autonomic nervous system. In a foreign language learning context, Horwitz et al. (1986) coined the term FLA and defined it as “a distinct complex of self-perceptions, beliefs, feelings, and behavior related to classroom language learning arising from the uniqueness of the language-learning process” (p. 28). Guo et al. (2018) considered Horwitz et al.’s (1986) Foreign Language Classroom Anxiety Scale (FLCAS) as a principal instrument for measuring FLA. To avoid the negative impacts of FLA on learners, numerous studies have been done to diagnose the reasons for FLA and suggest practical educational approaches to reduce its stimulation (Guo et al., 2018). Some strategies such as the length approach (Wang, 2005), cooperative learning techniques (Nagahashi, 2007), and applied psycho-social training (Kralova et al., 2017) were offered to diminish FLA in educational contexts. Horwitz et al. (1986) categorized FLA construct into test anxiety, fear of negative evaluation, and communication apprehension. Cakici (2016) stated that test anxiety is concerned with a conscious or unconscious fear of failing that learners experience in educational assessments and tests. Moreover, Downing et al. (2020) defined fear of negative evaluation as “being afraid of their negative evaluations, and thinking that they will give others negative information about themselves” (p. 2). On the other hand, commutation apprehension has been defined as avoiding communications with others (Bourhis and Allen, 1992). It has a significant correlation with language learners’ linguistic background and proficiency levels (Molnar and Crnjak, 2018). Rashidi et al. (2012) investigated English as a foreign language (EFL) learners’ communication apprehension, and they attributed it to the level of proficiency in language learners as beginners have a higher level of communication apprehension. FLA has also been widely considered a leading factor in academic achievement and language proficiency (MacIntyre, 2017). Studies have testified that higher anxiety levels are negatively correlated with foreign language proficiency, and with positive orientation and peer emotional support (Zheng and Cheng, 2018). Horwitz (2017) also found out that FLA has a significant negative correlation with some affective factors such as learners’ motivation, willingness to communicate, and self-esteem. COVID-19 pandemic affected learners’ FLA in online classrooms. Mohammed and Mudhsh (2021), in their study, revealed that COVID-19 pandemic causes normal anxiety to the EFL learners. They found out that there is no significant difference between the male and female learners in terms of anxiety caused by COVID-19 and external factors. Valizadeh (2021) investigated FLA in virtual classrooms during the Covid-19 pandemic in Turkey. The Turkish EFL university learners’ anxious feelings in traditional and virtual classrooms were compared in his study. He found out that the online classroom setting made a large number of the participants feel more suffocated and isolated.

### The Role of Online and Traditional Learning Context in English as a Foreign Language Learners’ Grit

The effect of educational contexts, such as face-to-face and online language learning, on learning achievement and performance has frequently been investigated by academic scholars (Chiu and Hew, 2018; Chien et al., 2020; Wahab and Iskandar, 2020). Jia (2004) asserted that face-to-face classroom learning, called traditional or offline learning, is a dynamic development of appealing and effective experiences to construct mental models of the world. He asserted that traditional classrooms provide an opportunity for learners to have face-to-face interaction between learners and instructors, which supports rapport among them. He maintained that encouraging classroom contexts for learners should be important for instructors to simplify learning. Nowadays, online language learning has been developed in order to meet the needs of learners in the world (Bolliger and Armier, 2013). Hurlbut (2018) stated that technology has markedly changed the learning and teaching contexts. There are numerous advantages to online learning such as increased opportuneness, flexibility, feedback, and interaction (Hsu et al., 2012); however, online learning contexts offer cognitive and psychological challenges for both instructors and students’ (Guri-Rosenblit, 2018).

Few studies have been done on the role of grit in learners’ performance in online educational contexts. For instance, Aparicio et al. (2017), in their study, found out that learners’ grit in online learning is significantly correlated with their satisfaction and performance. McClendon et al. (2017) listed some factors for learner engagement in online language classrooms. They stated that some non-cognitive factors, such as grit, persistence, and mindsets are significantly correlated with learners’ scores in online language learning. They argued that grit, mindset, and persistence increase learners’ engagement in online classes and improve learner achievement. Lan and Moscardino (2019) argued that grit was regarded as an essential component of educational success in a COVID-19 pandemic, and it was considered a psychological procedure that triggered and guided individuals’ activities in online learning contexts. Elahi Shirvan et al. (2021) investigated teacher’s fluctuations of effort and interest and how challenges may trigger the changes of effort and interest during the COVID-19 pandemic. Despite the fact that the teacher scored high on the grit scale, they found that the sudden shift from in-person to online teaching had put much pressure and demand on the teacher. Malureanu et al. (2021) explored the relationship among the individuals’ beliefs regarding self-confidence, grit, ease of use, self-efficacy, and usefulness of eLearning platforms during the COVID-19 pandemic. They found out that grit, self-efficacy, and perceived ease of use of eLearning platforms were considerably directly influenced by the self-confidence variable. Mediation analysis indicated that full mediation occurs only through the ease of use of eLearning platforms variable in the relationship between self-confidence and usefulness. Yang (2021) explored the relationship between Chinese EFL students’ grit, wellbeing, and classroom enjoyment in online classrooms during the COVID-19 pandemic. They found out that there is a positive relationship between learners’ grit and enjoyment, and high degrees of enjoyment were interrelated to high degrees of grit. They found out that grit significantly predicted students’ wellbeing and was also a predictor of classroom enjoyment.

Nussbaum et al. (2020) have emphasized the role of critical thinking, creativity, heuristic evaluation, and grit in the development of online language learning contexts. Liu et al. (2021) studied grit in mobile learning contexts among EFL learners. They found that grit has a significant relationship with academic performance in mobile EFL learning. Li and Dewaele (2021) revealed that the general grit and the classroom context in online English classrooms are significantly correlated with FLA. To the best of our knowledge, no studies have been done on the role of educational contexts in EFL learners’ grit.

### The Role of Online and Traditional Learning Context in English as a Foreign Language Learners’ Foreign Language Anxiety

Studies have shown that FLA in traditional classroom contexts has been a significant emotional variable in learning, and it has negatively affected learners “achievement. Many studies have been done on learners” anxiety levels in traditional face-to-face learning in classroom contexts. However, few studies have been delved into learners’ FLA in online language learning. For instance, Shahi (2016) found out that online learning through social media can decrease learner anxiety, and offer a less stressful educational context. He argued that scaffolding and teaching about using e-learning applications are critical in the reduction of anxiety since scaffolding contributes learners to comprehend the tasks better. In contrast, Hurd (2007), in a study, revealed that FLA levels in online learning are correlated with some issues like lack of feedback and opportunities for speaking in online contexts. He also argued that learners’ low confidence in online learning contexts and the type of employed applications can be described as other reasons for FLA. Kock (2004), in a study, also revealed that online language learning increases learners’ cognitive load, communication uncertainty, and reduction in enthusiasm which negatively impact learners’ FLA. Using media naturalness theory, they argued that self-confident and self-regulated learners could enjoy online learning education. Moreover, Russell (2020) revealed that online language learners’ level of anxiety, during COVID-19 increases since learners are not supported in choosing their educational learning style. Doğan (2020) also investigated the effect of online and traditional learning on learners’ FLA. He found out that learners are inclined to be anxious in an online context. He found two levels of anxiety among learners: FLA and online learning anxiety. He argued that a difference between a learner’s anticipation, experience, and apprehension from technology among learners can justify the reason for learners’ FLA and online learning anxiety. Demetriou et al. (2021) explored the relationships between employed and unemployed learners’ FLA levels and their adaptableness to online learning during the lockdown. They found that employment is significantly correlated with online learning and anxiety levels. Chametzky (2019) also stated that face-to-face interaction in traditional classrooms as a natural discipline in foreign language learning is not as ambiguous as online language learning. Therefore, it is obvious to anticipate foreign language classroom anxiety in distance learning contexts more than traditional ones. Having studied the anxiety levels of advanced learners in traditional and online learning contexts, Pichette (2009) did not find any difference between the effects of these two contexts on learners’ anxiety levels. He argued that anxiety is not a reasonable factor for foreign language engagement in distance learning.

## Implications and Suggestions for Further Research

This review examined the related literature on the effect of traditional and online learning on learners’ grit FLA. This study verifies Kock’s (2011) media naturalness theory which states that online learning with applications impose hurdles to learners since they can increase learners’ cognitive loads, the uncertainty of contexts, and reduce learner enthusiasm. This review implicates that learners should be aware of grit and FLA in online language learning by controlling, adjusting, and regulating their feelings in both language learning contexts. Learners can not only develop their academic achievement but also increase their grit with persistence and academic engagement in face-to-face and face-to-screen learning contexts. Instructors are required to decrease learners’ FLA and disengagement, and they need to increase learners’ grittiness irrespective of educational problems in language learning environments to enrich L2 learning experiences. They can offer warming-up activities for educational contexts and brainstorm learners to decrease ambiguity in online contexts. They can provide patterns for appropriate oral contexts and communication strategies in order to avoid anxiety in online contexts. Teachers can manage the time of classrooms regularly, and they can give relevant feedback to learners. They should talk to learners about their reasons for anxiety in online contexts. The projects, lectures, conferences, and workshops put extra stress on learners in online and classroom environments. In order to lessen the pressure, familiarizing learners with questions of the tests can be helpful, and teachers can change their course assessment in online and traditional classrooms. Moreover, teachers can arouse learner grit by reading books like “How Children Succeed: Grit, Curiosity, and the Hidden Power of Character” in traditional and online contexts. Teachers can provide learners with some video files like TED videos in online and traditional classrooms, and they can discuss the effect of gritty, inspiring individuals on their performance. Therefore, learner empowerment is a great strategy to develop grit in online and traditional contexts. Teachers can provide models to learners by playing videos about studious individuals who are gritty and triumphant in their life despite their disabilities. Also, instructors should live grittily to be a model for language learners in face-to-face and face-to-screen learning contexts. They should talk about their perseverance against the problems that learners know exactly what teachers think about grit. L2 instructors should try to find materials to develop learners’ positive attitudes and motivation for increasing their positive affectivity like grit, and to reduce negative feelings such as foreign language and disengagement in their classes. Teachers can also provide enjoyable language learning tasks in online and traditional contexts to consolidate learning learners’ positive emotions in their minds. This can diminish learners’ cognitive load in online learning, and increase their concentration on language learning contexts. Furthermore, EFL teachers’ awareness about learners’ personality traits, grittiness, and anxiety may encourage teachers to be engaged enthusiastically in online and traditional learning contexts. Teachers should encourage learners to use social media to increase language skills.

Teacher educators and mentors can provide some techniques for teachers to improve learners’ grittiness. They can hold workshops in pre-service and in-service teacher courses to talk about the importance of grit in language learning. It is also suggested that teacher educators should highlight interaction tools, like mobile applications, which promote interaction based on the subject matter. They should emphasize the effectiveness of interaction materials for face-to-face learning and face-to-screen learning. They can provide adequate educational and technical information, and they offer techniques for decreasing learner anxiety. This review recommends that teacher educators should have a positive view toward teachers and learners and they should provide well-organized and inspiring teaching methodologies which can construct a positive context for language learning, and increase learners’ grit and engagement in the classroom. Thus, paying attention to learners’ grit and anxiety in both online and traditional learning may improve learners’ feelings of approval which may foster teacher’s rapport with learners and eventually arouse learners’ academic performance in different educational contexts. This review can also stimulate educational policymakers to consider EFL learners’ negative and positive emotions in online and traditional contexts. They can hold academic workshops to help teachers decrease communication apprehension among learners. They can provide internet-based facilities and positive learning contexts for increasing positive behaviors among learners. The importance of grit and anxiety makes advisors widen their programs to recognize learners’ reasons for having FLA, and the obstacles they face when they want to persist in their effort.

Future studies are necessary to validate the numerous measures of grittiness and FLA. Longitudinal studies are needed to examine the long-lasting effects of educational contexts on EFL learners’ grit and anxiety. Also, studies need to be done on learners’ grit in online and traditional contexts in numerous cultural backgrounds. Some investigations need to be done on learners’ mindsets and other positive emotions in online and traditional classrooms. Other investigations can also be done on the relationship between different components of grit, and classroom anxiety in foreign language learning contexts. Online learning for EFL learners might not be appropriate to all learners. Therefore, future research should be devoted to the effect of gender, socio-economic background, age, along with earlier online learning experience on learner grit and anxiety. Furthermore, the proficiency level of foreign language learners and its effect on the relationship between online learning and grit should be highlighted for the future. In addition, conducting case and phenomenological investigations provides a good starting point for a discussion on the reasons behind anxiety in online learning contexts.

## Author Contributions

The author confirms being the sole contributor of this work, and has approved it for publication.

## Conflict of Interest

The author declares that the research was conducted in the absence of any commercial or financial relationships that could be construed as a potential conflict of interest.

## Publisher’s Note

All claims expressed in this article are solely those of the authors and do not necessarily represent those of their affiliated organizations, or those of the publisher, the editors and the reviewers. Any product that may be evaluated in this article, or claim that may be made by its manufacturer, is not guaranteed or endorsed by the publisher.
